# Anuran species composition of Cancão Municipal Natural Park, Municipality of Serra do Navio, Amapá state, Brazil

**DOI:** 10.3897/zookeys.762.22634

**Published:** 2018-05-31

**Authors:** Yuri Breno Silva e Silva, Carlos Eduardo Costa-Campos

**Affiliations:** 1 Universidade Federal do Amapá, Departamento de Ciências Biológicas e da Saúde, Laboratorio de Herpetologia, Rod. Juscelino Kubitschek, km 02, Jardim Marco Zero, CEP 68.903-419, Macapá, AP, Brasil

**Keywords:** Amazonia, conservation, eastern Amazon, species list

## Abstract

In this study, the first survey of anuran species in the Cancão Municipal Natural Park is presented, a protected area of approximately 370 hectares of Amazonian forest located in the northwest center region of the state of Amapá, Brazil. The work was performed during the dry and rainy season, through active visual and auditory survey, totaling 216 man hours of sampling effort. Forty-nine species of anuran amphibians were recorded in the Cancão Municipal Natural Park, including three new records: *Hyalinobatrachium iaspidiense*, Pristimantis cf. ockendeni, and *Scinax garbei*. Three species, *Hyalinobatrachium iaspidiense*, *Ameerega pulchripecta*, and *Anomaloglossus baeobatrachus*, are listed as Data Deficient and one is listed as Vulnerable (*Atelopus hoogmoedi*) according red lists of IUCN. The rarefaction curve cumulative species did not reach an asymptote, indicating that site has potential for species that have not yet been recorded. Nine species were represented by only one individual and were considered rare in the studied environments, eight species were defined as common, and the 32 remaining species were classified as having intermediary abundance. Our data indicated that Cancão Municipal Natural Park contains a considerable portion of the anurans species richness of Amapá state, turn the area into a place of great importance for the conservation of the anurans of the Eastern Amazon.

## Introduction

Most of the currently documented amphibian species in Brazil have been discovered during the last forty years (Campos et al. 2014). These new species descriptions, which have occurred at regular rates, are a strong indication that the Brazilian amphibian fauna is poorly known (Peloso 2010). Brazil has the highest diversity of amphibian species on the planet with 1080 species, 1039 of which are anurans, 36 caecilians, and five salamanders (Segalla et al. 2016). According to a recent publication of species list, 308 species of anurans (29.6 % of the species known in Brazil), 18 gymnophionans and five caudates (Hoogmoed and Galatti 2016) are known in the Brazilian Amazon, representing approximately one-third of the total of amphibians recorded for the country (Ávila-Pires et al. 2010, Neckel-Oliveira et al. 2013).

This amphibian species richness can be considered underestimated in number and complexity when considering enormous areas of Brazil which have yet to be inventoried, and there are many localities were surveys have been insufficient (Silvano and Segalla 2005). Aditionaly, the political limits and geographic distributions, the existence of cryptic species (Fouquet et al. 2007), sampling gaps due to the concentration of researches in a few areas (Azevedo-Ramos and Galatti 2002), sampling effort used appropriate methods for inventories of amphibians (Miranda et al. 2015) and problems in various taxonomic groups, frustrate attempts to obtain a comprehensive understanding of Brazil amphibians (Silvano and Segalla 2005).

Due to difficult to access, many Amazonian areas are still poorly known regarding their amphibian fauna and with insufficient sampling (Funk et al. 2012). In the Brazilian Amazonia, knowledge has increased in the last ten years based on studies on anurans composition conducted mostly in the state of Amazonas (França and Venâncio 2010, Ilha and Dixo 2010, Pantoja and Fraga 2012, Prudente et al. 2013, Waldez et al. 2013, Ferrão et al. 2016, Ferreira et al. 2017), state of Pará (Ávila-Pires et al. 2010, Mendes-Pinto and Souza 2011, Bernardo et al. 2012, Vaz-Silva et al. 2015), state of Rondônia (Ávila-Pires et al. 2010, Piatti et al. 2012) and state of Acre (Bernarde et al. 2011, Bernarde et al. 2013, Miranda et al. 2015, Venâncio and Souza 2016, França et al. 2017).

In the Amazonian biome, studies on anurans are concentrated in states of Amazonas, Pará, Rondônia and Acre, other localities in the Brazilian Amazon lack inventories (Azevedo-Ramos and Galatti 2002), a fact observed for the Amapá state. Although be inserted in a region of great interest for conservation and presents gaps knowledge on anurans (Azevedo-Ramos and Galatti 2002), Amapá state is little studied in relation to its anurans fauna (Lima 2008, Queiroz et al. 2011, Pereira-Júnior et al. 2013, Araújo and Costa-Campos 2014, Costa-Campos 2015, Costa-Campos et al. 2015, Lima et al. 2017, Benício and Lima 2017). In this context, the present study aims to provide the list of species of anuran amphibians that occur in the area of the Cancão Municipal Natural Park, municipality of Serra do Navio, state of Amapá, eastern Amazon.

## Materials and methods

**Figure 1.**
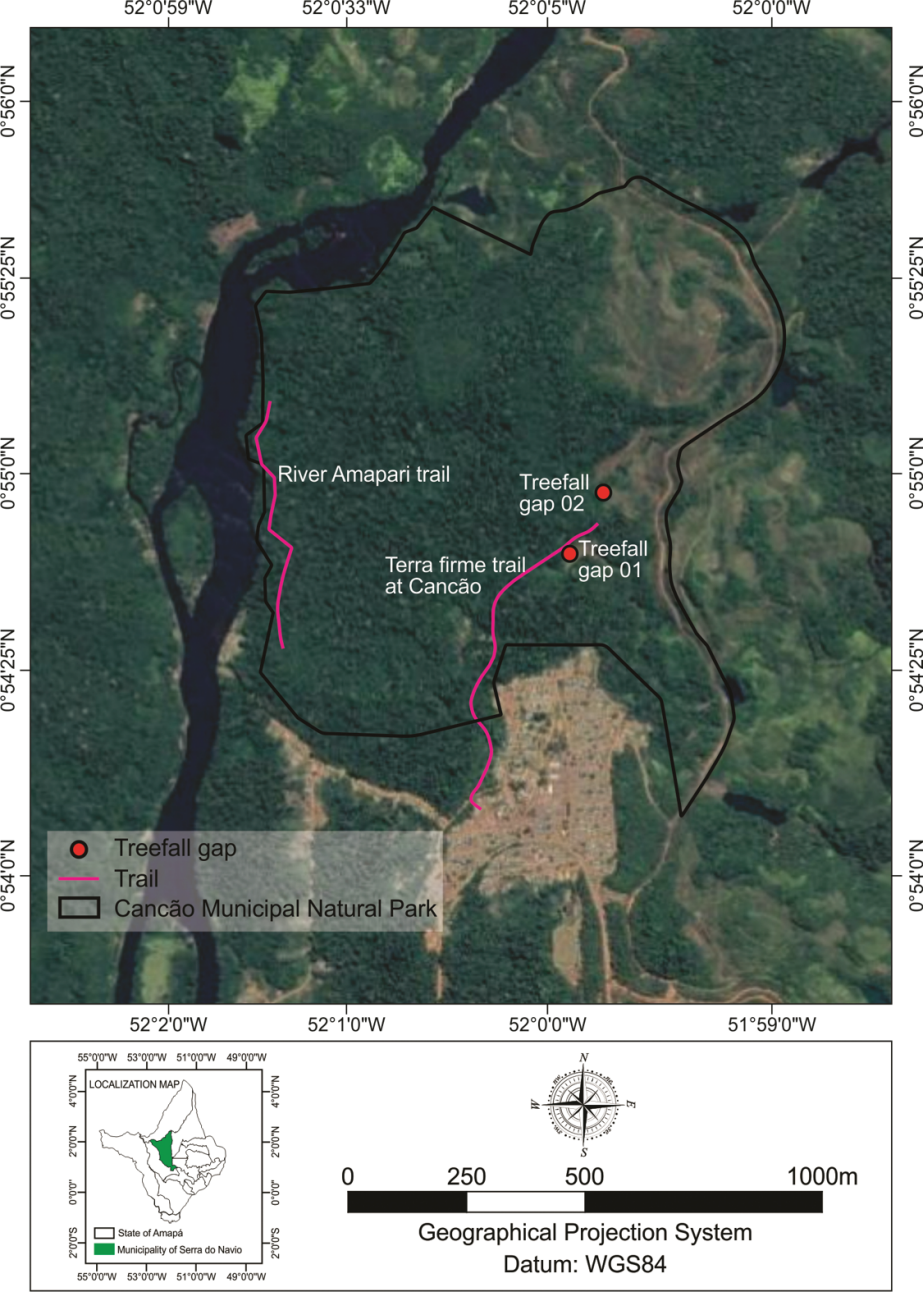
Maps showing the Amapá state and sampling sites in the Cancão Municipal Natural Park, municipality of Serra do Navio, Amapá State, northern Brazil.

*Study area*. Fieldwork was conducted at the Cancão Municipal Natural Park (Figure 1), municipality of Serra do Navio, Amapá state (0.90263°N, 52.00505°W and 0.90858°N, 52.00422°W). The study area covers approximately 370 hectares of primary forest, including terra-firme rainforests, streams, open areas, and treefall gaps. The climate of the region is Equatorial (Am) according Köppen-Geiger classification and the average temperature is 27.6 °C, varying seasonally between 25.8 to 29.0 °C, with annual rainfall approximately 2,850 mm with monsoon period between February and May, when the monthly rainfall is nearly 400 mm (Alvares et al. 2013).

**Figure 2.**
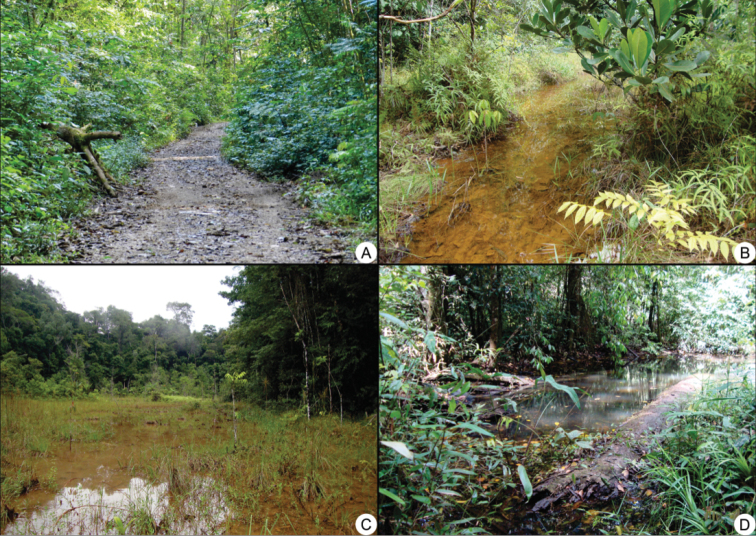
Habitats sampled at the Cancão Municipal Natural Park, municipality of Serra do Navio, Amapá state: **A** Terra firme trail at Cancão forest **B** Treefall gap at stream Cancão 01 **C** Treefall gap at Cancão forest 02 **D** River Amapari trail.

*Sampling*. Animals were registered during diurnal and nocturnal active visual search and auditory census in different microhabitats used by frogs (Heyer et al. 1994). These methods were conducted by three researchers for three consecutive days from January to December 2013, resulting in a sampling effort of 216 hours/man. A wide variety of environments were surveyed including ponds, brooks, forest interior, temporary ponds, and other water bodies. These environments were sampled in four mainly sites in the Cancão Municipal Natural Park (Figure 2): Terra firme trail at Cancão forest (0.90275°N, 52.00497°W); River Amapari trail (0.90083°N, 52.01347°W), Treefall gap at stream Cancão 01 (0.91183°N, 52.00205°W) and Treefall gap at Cancão forest 02 (0.91388°N, 51.99977°W).

The specimens were collected under permit SISBIO number 32651-1 issued by the Brazilian Ministry of Environment (MMA-ICMBio). Voucher adults collected were deposited at the Coleção Herpetológica da Universidade Federal do Amapá (UNIFAP) and Coleção Herpetológica do Museu Paraense Emílio Goeldi “Osvaldo Rodrigues da Cunha” (MPEG). The conservation status quoted follows IUCN (2017). The species taxonomy applied follows the Brazilian Society of Herpetology (SBH), according to Segalla et al. (2016) and Dubois (2017). *Adenomera andreae* and *A. hylaedactyla* were identified through morphology and vocalization (cf. Heyer 1973; Angulo et al. 2003).

*Data analysis*. To analyze the anurans species richness, rarefaction curves of species were constructed based on the number of individuals and number of samples (Gotelli and Colwell 2001) with 1000 randomizations. Species richness estimators Jacknife1 and Bootstrap were used for determine the expected richness of anurans (Colwell 2013). To determine similarities of species compositions amongst habitats sampled, cluster analyses were performed by the UPGMA method, using the modified index of similarity of Jaccard (Clarke 2003). This analysis was performed using ESTIMATES 9.1 (Gotelli and Colwell 2001).

The dominances were represented by Whittaker Diagram, obtained by ranking species, starting with the most abundant, along the x-axis and the logarithm abundances on the y-axis. Rare species were those represented by a single individual (singletons). The other species were classified as having intermediate abundance. The pattern of the species abundance distribution was fitted to the geometric, logarithmic, log-normal, and broken-stick models. Model fit was assessed by the chi square adherence test (Magurran 2011) using the software PAST version 2.17c (Hammer et al. 2001).

The Spearman correlation coefficient analysis was performed to compare climatic conditions (available from the NHMET database) during the sampling period with abundance. To check the influence of environmental data on amphibian abundance, multiple regression analyses were conducted, including data on rainfall, temperature, and humidity as independent variables. The normality of the data was tested with the KOLMOGOROV-SMIRNOV analysis (Zar 1999). Statistical analyses were performed with BIOESTAT 5.3 software (Ayres et al. 2007), using a significance index of P < 0.05 for all analyses.

## Results

Forty-nine anuran species have been recorded in the Cancão Municipal Natural Park (Table 1, Figure 3) during the dry and rainy season, totaling 216 hours of sample effort. These species are distributed in 22 genera, belonging to five families: Allophrynidae (1 species); Aromobatidae (2 species), Bufonidae (5 species), Centrolenidae (1 species), Craugastoridae (5 species), Dendrobatidae (2 species), Eleutherodactylidae (1 species); Hylidae (18 species), Leptodactylidae (10 species), Phyllomedusidae (3 species); Pipidae (1 species).

**Figure 3.**
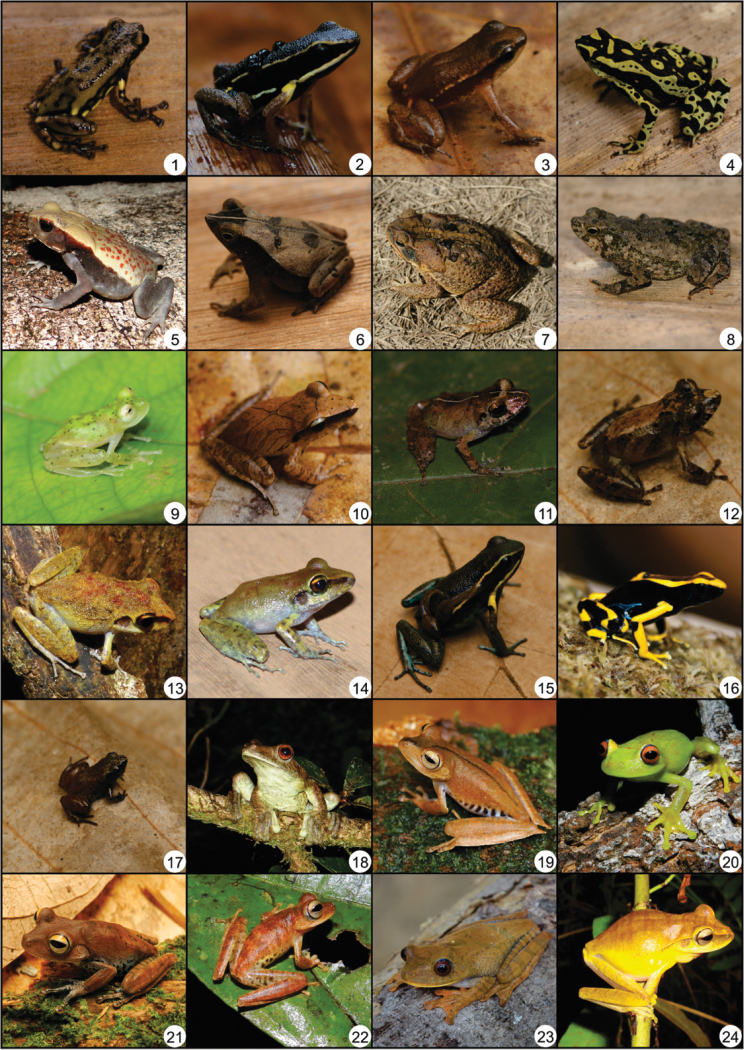
Species recorded in the Cancão Municipal Natural Park, municipality of Serra do Navio, Amapá state: **1** *Allophryne ruthveni* **2** *Allobates femoralis* **3** *Anomaloglossus baeobatrachus* **4** *Atelopus hoogmoedi* **5** *Rhaebo guttatus* **6** *Rhinella margaritifera* complex **7** *R. marina* **8** *R. martyi* **9** *Hyalinobatrachium iaspidiense* **10** *Pristimantis chiastonotus* **11** *P. marmoratus* **12** P. cf. ockendeni **13** *P. zeuctotylus* **14** *P. zimmermanae* **15** *Ameerega pulchripecta* **16** *Dendrobates tinctorius* **17** *Adelophryne gutturosa* **18** *Boana boans* **19** *B. calcarata* **20** *B. cinerascens* **21** *B. dentei* **22** *B. fasciata* **23** *B. geographica* **24** *B. multifasciata*.

**Figure 3.**
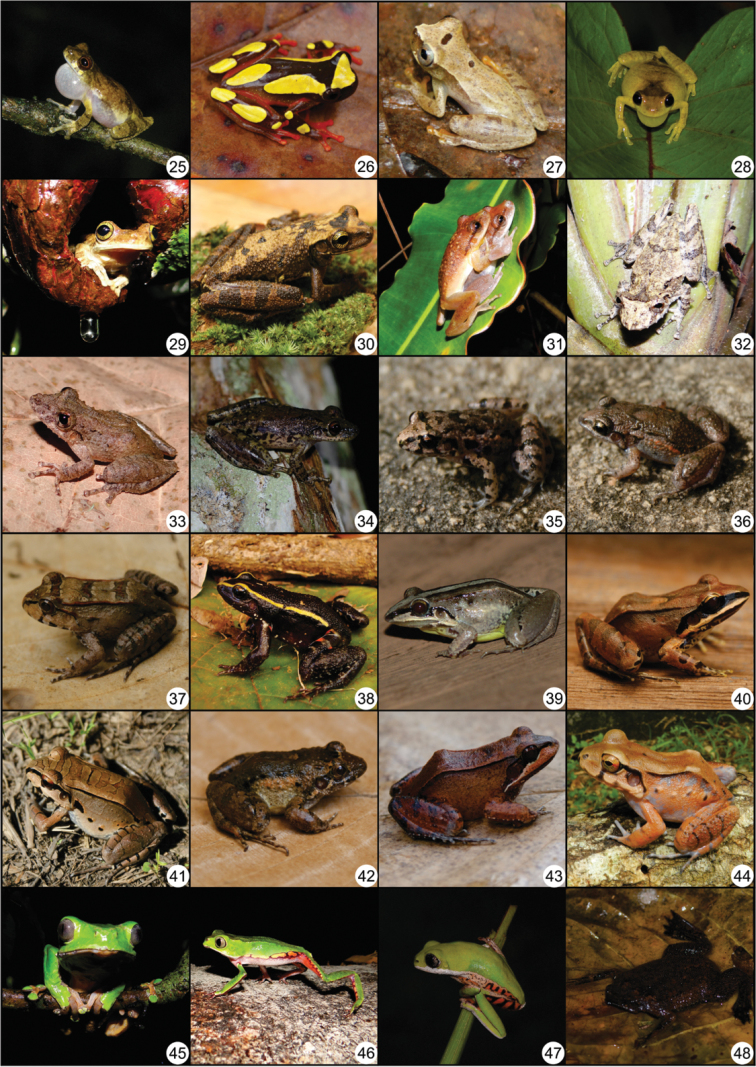
Continued. **25** *Dendropsophus counani* **26** *D. leucophyllatus* **27** Dendropsophus cf. microcephalus **28** *D. minutus* **29** *Osteocephalus oophagus* **30** *O. taurinus* **31** *Scinax boesemani* **32** *S. garbei* **33** *S. nebulosus* **34** *S. ruber* **35** *Adenomera andreae* **36** *A. hylaedactyla* **37** *Leptodactylus knudseni* **38** *L. lineatus* **39** *L. longirostris* **40** *L. mystaceus* **41** *L. pentadactylus* **42** *L. petersii* **43** *L. rhodomystax* **44** *L. stenodema* **45** *Phyllomedusa bicolor* **46** *P. vaillantii* **47** *Pithecopus hypochondrialis*, and **48** *Pipa pipa*.

**Table 1.** List of amphibian species recorded at Cancão Municipal Natural Park, municipality of Serra do Navio, Amapá State. Sampled areas: Terra firme trail at Cancão forest (TC), Amapari trail (TA), treefall gap at stream Cancão (TS), and treefall gap at Cancão forest (TF). Red List species included in some category of IUCN (2017): LC – Least Concern; VU – Vulnerable; DD – Data Deficient.

Family/Species	Sampled areas				IUCN
	TC	TA	TS	TF	
**Allophrynidae**					
*Allophryne ruthveni* Gaige, 1926		X			LC
**Aromobatidae (Allobatinae)**					
*Allobates femoralis* (Boulenger, 1884)	X	X			LC
**Aromobatidae (Aromobatinae)**					
*Anomaloglossus baeobatrachus* (Boistel & de Massari, 1999)				X	DD
**Bufonidae**					
*Atelopus hoogmoedi* Lescure, 1974		X			VU
*Rhaebo guttatus* (Schneider, 1799)	X	X			LC
*Rhinella margaritifera* complex of species	X	X	X	X	LC
*Rhinella marina* (Linnaeus, 1758)	X	X	X	X	LC
*Rhinella martyi* Fouquet, Gaucher, Blanc & Vélez-Rodriguez, 2007		X			LC
**Centrolenidae (Hyalinobatrachinae)**					
*Hyalinobatrachium iaspidiense* (Ayarzaqüena, 1992) *		X			DD
**Craugastoridae (Ceuthomantinae)**					
*Pristimantis chiastonotus* (Lynch & Hoogmoed, 1977)	X	X		X	LC
*Pristimantis marmoratus* (Boulenger, 1900)		X			LC
Pristimantis cf. ockendeni (Boulenger, 1912) *	X	X		X	LC
*Pristimantis zeuctotylus* (Lynch & Hoogmoed, 1977)		X			LC
*Pristimantis zimmermanae* (Heyer & Hardy, 1991)		X			LC
**Dendrobatidae (Colostethinae)**					
*Ameerega pulchripecta* (Silverstone, 1976) **	X	X			DD
**Dendrobatidae (Dendrobatinae)**					
*Dendrobates tinctorius* (Cuvier, 1797)		X			LC
**Eleutherodactylidae**					
*Adelophryne gutturosa* Hoogmoed & Lescure, 1984	X				LC
**Hylidae**					
*Boana boans* (Linnaeus, 1758)	X	X	X	X	LC
*Boana calcarata* (Troschel in Schomburgk 1848)		X			LC
*Boana cinerascens* (Spix, 1824)	X		X	X	LC
*Boana dentei* (Bokermann, 1967) **		X			LC
*Boana fasciata* (Günther, 1859 “1858”)		X		X	LC
*Boana geographica* (Spix, 1824)		X			LC
*Boana multifasciata* (Günther, 1859 “1858”)	X		X	X	LC
*Dendropsophus counani* Fouquet, Souza, Nunes, Kok, Curcio, de Carvalho, Grant & Rodrigues, 2015	X	X			–
*Dendropsophus leucophyllatus* (Beireis, 1783)				X	LC
Dendropsophus cf. microcephalus (Cope, 1886)	X				LC
*Dendropsophus minutus* (Peters, 1872)	X				LC
*Osteocephalus oophagus* Jungfer & Schiesari, 1995		X			LC
*Osteocephalus taurinus* Steindachner, 1862	X	X			LC
*Scinax boesemani* (Goin, 1966)			X	X	LC
*Scinax garbei* (Miranda-Ribeiro, 1926) *				X	LC
*Scinax nebulosus* (Spix, 1824)			X	X	LC
*Scinax ruber* (Laurenti, 1768)	X	X	X	X	LC
*Trachycephalus resinifictrix* (Goeldi, 1907)	X	X			LC
**Leptodactylidae (Leptodactylinae)**					
*Adenomera andreae* (Müller, 1923)	X	X	X	X	LC
*Adenomera hylaedactyla* (Cope, 1868)	X	X	X	X	LC
*Leptodactylus knudseni* Heyer, 1972	X				LC
*Leptodactylus lineatus* (Schneider, 1799)	X				LC
*Leptodactylus longirostris* Boulenger, 1882		X	X	X	LC
*Leptodactylus mystaceus* (Spix, 1824)	X	X	X	X	LC
*Leptodactylus pentadactylus* (Laurenti, 1768)	X				LC
*Leptodactylus petersii* (Steindachner, 1864)	X				LC
*Leptodactylus rhodomystax* Boulenger, 1884 “1883”	X				LC
*Leptodactylus stenodema* Jiménez de la Espada, 1875	X				LC
**Phyllomedusidae**					
*Phyllomedusa bicolor* (Boddaert, 1772)		X			LC
*Phyllomedusa vaillantii* Boulenger, 1882	X	X			LC
*Pithecopus hypochondrialis* (Daudin, 1800)	X	X		X	LC
**Pipidae**					
*Pipa pipa* (Linnaeus, 1758)		X			LC

* First record for the state of Amapá. ** Species endemic to the municipality of Serra do Navio, Amapá state.

Three new records of anurans are presented for the Cancão Municipal Natural Park, namely *Hyalinobatrachium iaspidiense* (Centrolenidae), Pristimantis cf. ockendeni (Craugastoridae), and *Scinax garbei* (Hylidae). None of the frog species recorded at the Cancão Municipal Natural Park is classified as threatened in the red lists of IUCN (2017). However, three species (*Hyalinobatrachium iaspidiense*, *Ameerega pulchripecta*, and *Anomaloglossus baeobatrachus*) are listed as Data Deficient and one is listed as Vulnerable (*Atelopus hoogmoedi*).

*Hyalinobatrachium iaspidiense* is known from Brazil, Ecuador, French Guiana, Guyana, Peru, Suriname, Venezuela, and is expected to occur in the Amazonian areas between the Ecuadorian and Peruvian localities and the Guiana region (Castroviejo-Fisher et al. 2011). This record is the first for Amapa and extends the known distribution of the species 1,020 km east from the type locality Quebrada de Jaspe, San Ignacio de Yuraní, Bolívar state, Venezuela (Silva e Silva and Costa-Campos 2016).

Pristimantis cf. ockendeni is distributed throughout the Amazonian basin of Peru, Ecuador, southern Colombia, and Brazil in the states of Acre and Amazonas (Rodríguez et al. 2004). This is the first state record for Amapa, extending the range 986 km NW from the Manaus, Amazonas state (Silva e Silva et al. 2015).

*Scinax garbei* is known from Ecuador, adjacent Peru, Bolivia, Colombia, and Venezuela (Frost 2018). In Brazilian Amazonia, it has been recorded from Amazonas (França and Venâncio 2010) and Pará states (Ávila-Pires et al. 2010). In this study, we present the first record of the species in the state of Amapá, extending the species distribution in the Brazilian Amazonia by 525 km northward from the two localities in the state of Pará: Rio Xingu and Rio Curuá-Una (Silva e Silva and Costa-Campos 2014).

**Figure 4.**
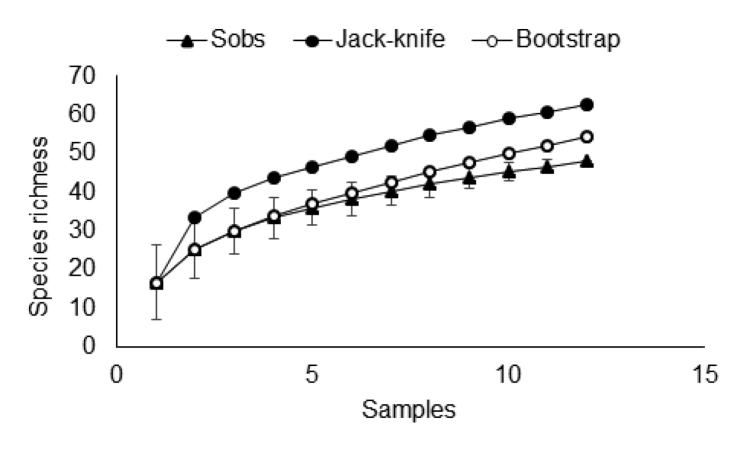
Rarefaction curve of anuran species based on the species records and sampling effort (sampling days) in the Cancão Municipal Natural Park, Amapá state, northern Brazil. Richness estimators used: Jack-knife 1 and Bootstrap. Sobs = total number of species observed in a samples.

The frog species richness estimated for the area by Bootstrap and Jack-knife was 54 and 63 species, respectively, and the rarefaction curve cumulative species did not reach an asymptote. We believe that site has potential for species that have not yet been recorded (Figure 4). The Hylidae was the most species-rich family (17 species), followed by the Leptodactylidae (10) and Craugastoridae (4).

**Figure 5.**
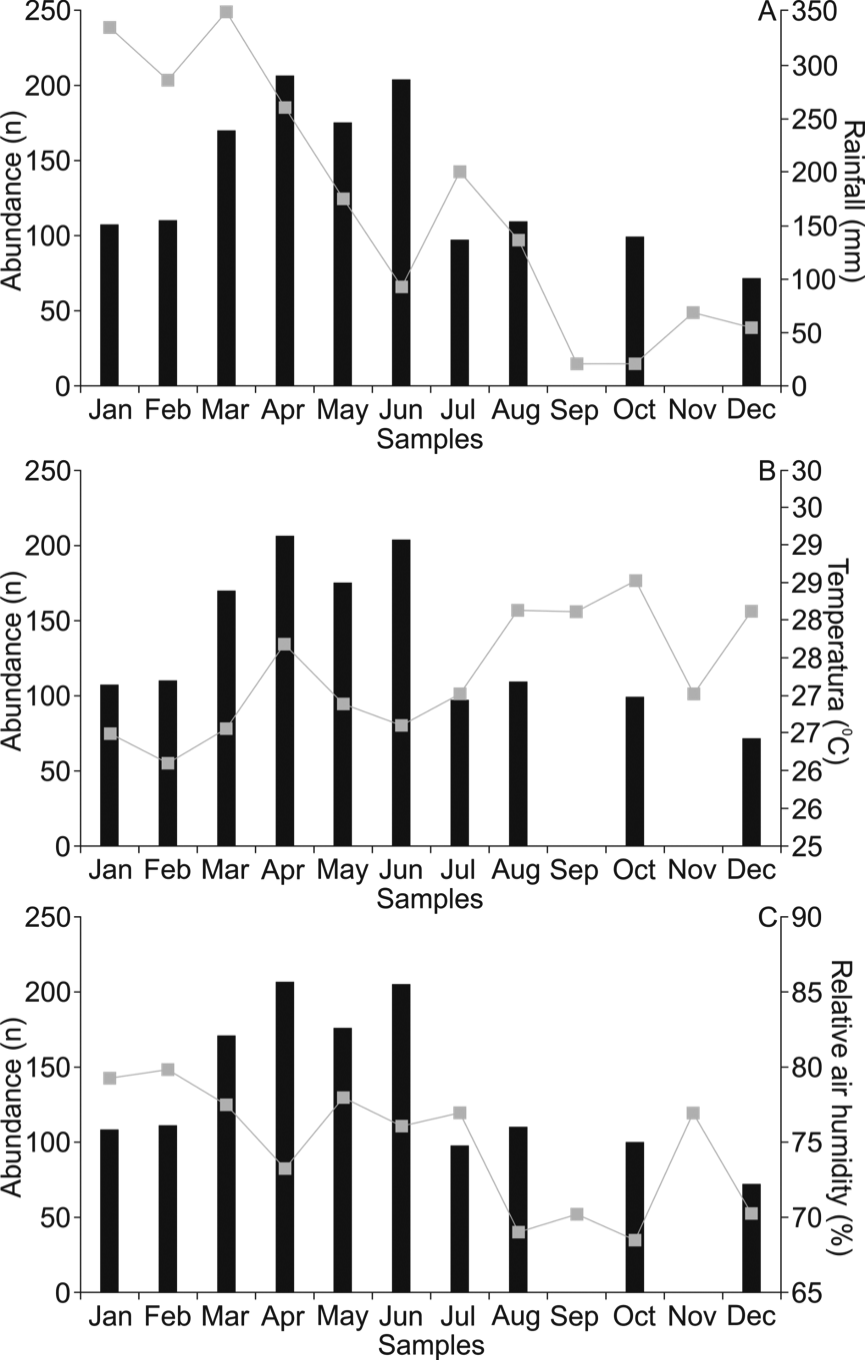
Correlation of recorded anuran abundance and abiotic factors in Cancão Municipal Natural Park, municipality of Serra do Navio Amapá state, from January through December 2013. **A** Anuran abundance (black bars) and rainfall (grey squares and line) **B** anuran abundance (black bars) and temperature (grey squares and line) **C** anuran abundance (black bars) and relative humidity (grey squares and line).

Spearman correlations obtained with the studied period were not significant for rainfall data (R = 0.564, P = 0.056), temperature (R = 0.467, P = 0.167) and relative humidity (R = 0.267, P = 0.877). According to multiple regressions, amphibian abundance does not seem to be related to any of the abiotic factors considered (F3, 8 = 1.240; P = 0.179; r = 0.563 for the entire analysis; F3, 8 = 3.422; P = 0.091 for rainfall; F3, 8 = 1.097; P = 0.320 for temperature; and F3, 8 = 0.720; P = 0.579 for relative humidity (Figure 5). With regard to total frog abundance, higher values were computed in rainy seasons compared to dry seasons.

**Figure 6.**
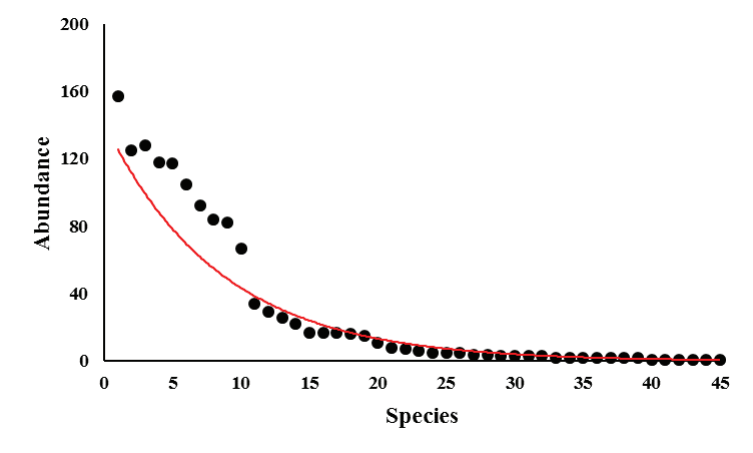
Whittaker diagram for the abundance distribution of amphibians recorded in the Cancão Municipal Natural Park, municipality of Serra do Navio, Amapá state.

Nine species (*Allophryne ruthveni*, *Rhinella martyi*, *Pristimantis zimmermanae*, *Boana calcarata*, *B. dentei*, *Osteocephalus oophagus*, *Scinax garbei*, *Leptodactylus lineatus* and *Pipa pipa*) were represented by only one individual and were considered rare in the studied environments. Applying the number of singletons to the other end of the abundance distribution, eight species were defined as common, including *P. chiastonotus* (157 individuals), *B. multifasciata* (123 individuals), *R. margaritifera* complex (123 individuals), and *B. boans* (105 individuals). The 32 remaining species were classified as having intermediary abundance (Figure 6).

**Figure 7.**
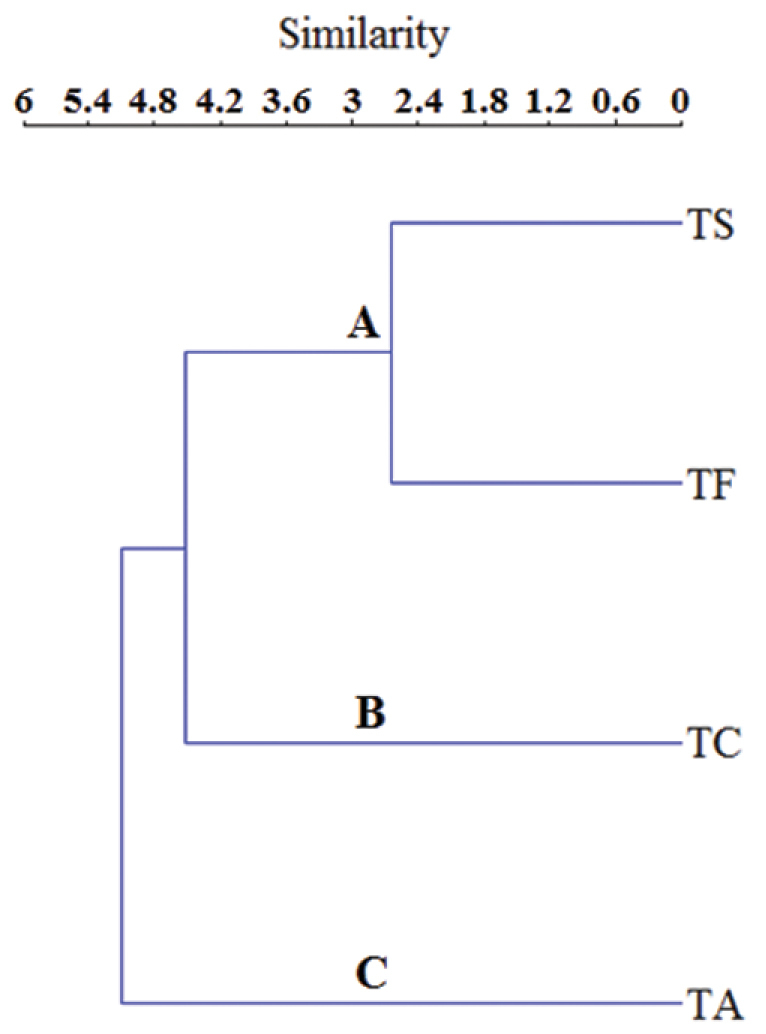
Dendrogram for cluster analysis (UPGMA) using the Jaccard’s Similarity Index between the anurans of the Cancão Municipal Natural Park. Cophenetic correlation coefficient = 0.997. Sampled areas: Group **A** Treefall gap at stream Cancão (TS) and Treefall gap at Cancão forest (TF); Group **B** Terra firme trail at Cancão forest (TC); Group **C** Amapari trail (TA).

The dendrogram obtained from cluster analysis evidences three major groups: (A) sites located in the treefall gaps, (B) sites in the Terra firme forest, and (C) sites belonging to the Amapari River with temporary ponds. The group (A) is characterized by the higher *Boana multifasciata*, *B. cinerascens* and *Pithecopus hypochondrialis*, species occurring in open areas. For the group (B), the most frequent species were *Rhinella margaritifera* complex species and *Pristimantis chiastonotus*. The last group, (C), is characterized by the high frequency of occurrence of *Allobates femoralis* and *Adenomera andreae*. The coefficient of cophenetic correlation for the cluster analysis was 0.997 (Figure 7).

## Discussion

Our data indicate that Cancão Municipal Natural Park contains a considerable portion of the anurans species richness of Amapá state. The anuran fauna corresponded to 65.7 % of the recorded species for the Tumucumaque Mountains National Park (Lima 2008), 86.9 % of the species found in the River Cajari Extractive Reserve (Queiroz et al. 2011), 60.9 % species recorded during the surveys conducted of the Rio Curiaú Environmental Protection Area (Lima et al. 2017), and 90.6 % species of anurans recorded in the Amapá National Forest (Bemício and Lima 2017). This high anurans richness for the Amazonian biome is highly underestimated considering taxonomic problems, recent descriptions of species and taxonomic revisions (Peloso et al. 2014; Vaz-Silva et al. 2015).

The results obtained from the rarefaction curve and the Jack-knife1 and Bootstrap estimators suggest that the species composition is still underestimated, and more long-term studies may reveal the presence of additional species in the area. Future studies should be complemented with combined and/or different approaches in fieldwork, such as the use of pitfall traps for leaf-litter species, increased visual search times (Freitas et al. 2017).

The finding of a large number of species of the families Hylidae and Leptodactylidae was similar to the results of other studies and follows the pattern found in neotropical environments (Segalla et al. 2016), including the Brazilian Amazon (Azevedo-Ramos and Galatti 2002, Neckel-Oliveira et al. 2013, Ramalho et al. 2016). In addition, three species of anurans (Pristimantis cf. ockendeni, *Hyalinobatrachium iaspidiense* and *Scinax garbei*) are new records in the Amapá state (Silva e Silva and Costa-Campos 2014, Silva e Silva et al. 2015, Silva e Silva and Costa-Campos 2016), evidence the incipience of knowledge in the regional context due to the lack of sampling.

The record of *Atelopus hoogmoedi* and *Ameerega pulchripecta* in the area studied is relatively important. *Atelopus hoogmoedi* is a terrestrial and diurnal species, and is most commonly found at small streams in primary forest (Ouboter and Jairam 2012). The species occurs in the Amazonian lowlands of Colombia, Ecuador, and eastern Peru, to Amazonas, Pará, Amapá (Brazil), and the Guianas (Frost 2018). *Ameerega pulchripecta* was hard to find, and it has been heard only during less than an hour around dawn and again around twilight (Costa-Campos et al. 2016). Additionally, its distribution appears restricted to Serra do Navio, in the state of Amapá, northeastern Brazilian Amazon. These species are classified as vulnerable and data deficient by the IUCN due to their areas of occurrence, status and little known ecological requirements (IUCN 2017).

The cluster analysis of the anuran assemblages generated three groups. The group A and B showed a more differentiated assemblage. Group C are located on the right bank of the Amapari River, and presented high values of abundance and richness. The results can be attributed to the similar characteristics between the sites. The main hypotheses proposed to explain barrier formation separating populations and causing the differentiation of species in Amazonia during the course of geological history are based on different factors (Haffer 2008). According the river hypothesis, rivers may play a major role in creating and maintaining high levels of spatial separation of populations (Vaz-Silva et al. 2015).

## Conclusions

The results of the present study thus provide new data on geographic distribution of species showed three new records of the Brazilian Amazonian and important insights into the diversity of amphibians in the northern Brazil. The high amphibian richness recorded in this study for the eastern Amazon, combined with the presence of populations of Data Deficient or Vulnerable species, contributes to the knowledge on species, reinforcing the importance of the Cancão Municipal Natural Park for the conservation of anurans species.

## References

[B1] Alvares CA Stape JL Sentelhas PC Gonçalves JLM Sparovek G (2013) Köppen’s climate classification map for Brazil. Meteorologische Zeitschrift 22(6): 711–728. https://doi.org/10.1127/0941-2948/2013/0507

[B2] Angulo A Cocroft RB Reichle S (2003) Species identity in the genus *Adenomera* (Anura: Leptodactylidae) in southeastern Peru. Herpetologica 59(4): 490–504. https://doi.org/10.1655/20-104

[B3] Araújo AS Costa-Campos CE (2014) Anurans of the Reserva Biológica do Parazinho, Municipality of Macapá, state of Amapá, eastern Amazon. Check List 10(6): 1414–1419. doi: http://dx.doi.org/10.15560/10.6.1414

[B4] Ávila-Pires TCS Hoogmoed MS Rocha WA (2010) Notes on the Vertebrates of northern Pará, Brazil: a forgotten part of the Guianan Region, I. Herpetofauna. Boletim do Museu Paraense Emílio Goeldi, Ciências Naturais 5: 13–112.

[B5] Ayres M Ayres Jr. M Ayres DL Santos AA (2007) Bioestat 5.3. Aplicações estatísticas nas áreas das ciências biomédicas. Ong Mamiraua, Belém, PA.

[B6] Azevedo-Ramos C Galatti U (2002) Patterns of Amphibian Diversity in Brazilian Amazonia: Conservation Implications. Biological Conservation 103(1): 103–111. https://doi.org/10.1016/S0006-3207(01)00129-X

[B7] Benício RA Lima JD (2017) Anurans of Amapá National Forest, Eastern Amazonia, Brazil. Herpetology Notes 10: 627–633.

[B8] Bernarde PS Albuquerque S Miranda DB Turci LCB (2013) Herpetofauna da floresta do baixo rio Moa em Cruzeiro do Sul, Acre – Brasil. Biota Neotropica 13(1): 220–244. https://doi.org/10.1590/S1676-06032013000100023

[B9] Bernarde PS Machado RA Turci LCB (2011) Herpetofauna da área do Igarapé Esperança na Reserva Extrativista Riozinho da Liberdade, Acre – Brasil. Biota Neotropica 11(3): 117–144. https://doi.org/10.1590/S1676-06032011000300010

[B10] Bernardo PH Guerra-Fuentes RA Matiazzi W Zaher H (2012) Checklist of Amphibians and Reptiles of Reserva Biológica do Tapirapé, Pará, Brazil. Check List 8(5): 839–846. https://doi.org/10.15560/8.5.839

[B11] Campos FS Brito D Solé M (2014) Diversity patterns, research trends and mismatches of the investigative efforts to amphibian conservation in Brazil. Anais da Academia Brasileira de Ciências 86(4): 1873–1886. http://dx.doi.org/10.1590/0001-3765201420140170 25590723 10.1590/0001-3765201420140170

[B12] Castroviejo-Fisher S Vilà C Ayarzagüena J Blanc M Ernst R (2011) Species diversity of *Hyalinobatrachium* glassfrogs (Amphibia: Centrolenidae) from the Guiana Shield, with the description of two new species. Zootaxa 3132: 1–55.

[B13] Clarke KR (1993) Non-parametric multivariate analysis of changes in community structure. Austral Ecology 18: 117–143. https://doi.org/10.1111/j.1442-9993.1993.tb00438.x

[B14] Colwell RK (2013) EstimateS: Statistical estimation of species richness and shared species from samples. Version 9. http://purl.oclc.org/estimates [accessed 12 March 2017]

[B15] Costa-Campos CE (2015) Ecologia de comunidade e comportamento reprodutivo de anfíbios anuros em Savana Amazônica. Tese de Doutorado, Natal, Rio Grande do Norte: Universidade Federal do Rio Grande do Norte.

[B16] Costa-Campos CE Lima JD Reis JRF (2015) Riqueza e composição de répteis Squamata (lagartos e anfisbenas) da Área de Proteção Ambiental da Fazendinha, Amapá, Brasil. Biota Amazônia 5(2): 84–90. https://doi.org/10.18561/2179-5746/biotaamazonia.v5n2p84-90

[B17] Costa-Campos CE. Lima AP Amézquita A (2016) The advertisement call of *Ameerega pulchripecta* (Silverstone, 1976) (Anura, Dendrobatidae). Zootaxa 4136(2): 387–389. http://doi.org/10.11646/zootaxa.4136.2.9 27395723 10.11646/zootaxa.4136.2.9

[B18] Dubois A (2017) The nomenclatural status of *Hysaplesia*, *Hylaplesia*, *Dendrobates* and related nomina (Amphibia, Anura), with general comments on zoological nomenclature and its governance, as well as on taxonomic databases and websites. Bionomina 11: 1–48. https://doi.org/10.11646/bionomina.11.1.1

[B19] Ferrão M Colatreli O de Fraga R Kaefer IL Moravec J Lima AP (2016) High Species Richness of *Scinax* Treefrogs (Hylidae) in a Threatened Amazonian Landscape Revealed by an Integrative Approach. PLoS ONE 11 (11): e0165679. https://doi.org/10.1371/journal.pone.0165679 10.1371/journal.pone.0165679 PMC5091857 27806089

[B20] Ferreira GC Sturaro MJ Peloso PLV (2017) Amphibians and reptiles from Floresta Nacional de Pau-Rosa, Amazonas, Brazil: an important protected area at the heart of Amazonia. Acta Amazônia 47(3): 259–268. http://dx.doi.org/10.1590/1809-4392201602982

[B21] Fouquet A Vences M Salducci MD Meyer A Marty C Blanc M Gilles A (2007) Revealing cryptic diversity using molecular phylogenetics and phylogeography in frogs of the *Scinax ruber* and *Rhinella margaritifera* species groups. Molecular Phylogenetics and Evolution 43: 567–582. https://doi.org/10.1016/j.ympev.2006.12.006 17303441 10.1016/j.ympev.2006.12.006

[B22] França FGR Venâncio NM (2010) Reptiles and amphibians of a poorly known region in southwest Amazonia. Biotemas 23(3): 71–84. http://dx.doi.org/10.5007/2175-7925.2010v23n3p71

[B23] França DPF Freita MA Ramalho WP Bernarde OS (2017) Local diversity and influence of seasonality on amphibians and reptiles assemblages in the Reserva Extrativista Chico Mendes, Acre, Brazil. Iheringia, Série Zoologia 107: e2017023. http://dx.doi.org/10.1590/1678-4766e2017023

[B24] Freitas MA Vieira RS Entiauspe-Neto OM Oliveira e Sousa S Farias T Sousa AG Moura GJB (2017) Herpetofauna of the Northwest Amazon forest in the state of Maranhão, Brazil, with remarks on the Gurupi Biological Reserve. ZooKeys 643: 141–155. https://doi.org/10.3897/zookeys.643.8215 10.3897/zookeys.643.8215 PMC5242272 28144181

[B25] Frost DR (2018) Amphibian Species of the World: an Online Reference. Version 6.0. http://research.amnh.org/herpetology/amphibia/index.html [accessed on 15 January 2018]

[B26] Funk WC Caminer M Ron SR (2012) High level of cryptic species diversity uncovered in Amazonian frogs. Proceedings of the Royal Society B: Biological Sciences 279: 1806–1814. https://doi.org/10.1098/rspb.2011.1653 22130600 10.1098/rspb.2011.1653 PMC3297442

[B27] Gotelli NJ Colwell RK (2001) Quantifying biodiversity: procedures and pitfalls in the measurement and comparison of species richness. Ecology Letters 4(4): 379–391. https://doi.org/10.1046/j.1461-0248.2001.00230.x

[B28] Haffer J (2008) Hypotheses to explain the origin of species in Amazonia. Brazilian Journal of Biology 68(4): 917–47. https://doi.org/10.1590/S1519-69842008000500003 10.1590/s1519-69842008000500003 19197466

[B29] Hammer O Harper DAT Ryan PD (2001) PAST: Paleontological Statistics Software Package for Education and Data Analysis. Palaeontologia Electronica 4(1): 9. http://palaeo-electronica.org/2001_1/past/issue1_01.htm

[B30] Heyer WR (1973) Systematics of the *marmoratus* group of the frog genus *Leptodactylus* (Amphibia, Leptodactylidae). Contributions in Science, Natural History Museum, Los Angeles County 251: 1–50.

[B31] Heyer WR Donnelly MA McDiarmid RW Hayek LAC Foster MS (1994) Measuring and monitoring biological diversity: standard methods for amphibians. Smithsonian Institution Press, Washington, 384 pp.

[B32] Hoogmoed MS Galatti U (2016) Censo da Biodiversidade da Amazônia Brasileira. Grupo: Anura http://www.museu-goeldi.br/censo [accessed on 25 March 2016]

[B33] Ilha P Dixo M (2010) Anurans and Lizards, Rio Preto da Eva, Amazonas, Brazil. Check List 6(1): 17–21. https://doi.org/10.15560/6.1.017

[B34] IUCN (2017) IUCN Red List of Threatened Species. Version 2015.1. http://www.iucnredlist.org/ [accessed on 25 February 2017]

[B35] Lima JD (2008) A herpetofauna do Parque Nacional Montanhas do Tumucumaque, Amapá, Brasil, Expedições I a V. In: Bernard E (Ed.) Inventários Biológicos Rápidos no Parque Nacional Montanhas do Tumucumaque, Amapá, Brasil. RAP Bulletin of Biological Assessment 48. Conservation International, Arlington, 38–50.

[B36] Lima JRF Lima JDL Lima SD Lima-Silva RB Andrade GV (2017) Amphibians found in the Amazonian Savanna of the Rio Curiaú Environmental Protection Area in Amapá, Brazil. Biota Neotropica 17(2): e20160252. http://dx.doi.org/10.1590/1676-0611-BN-2016-0252

[B37] Magurran AE (2011) Medindo a diversidade biológica. Editora UFPR, Curitiba, 261 pp.

[B38] Mendes-Pinto TJ Souza SM (2011) Preliminary assessment of amphibians and reptiles from Floresta Nacional do Trairão, with a new snake record for the Pará state, Brazilian Amazon. Salamandra 47(4): 199–206.

[B39] Miranda DB Albuquerque S Turci LCB Bernarde PS (2015) Richness, breeding environments and calling activity of the anurofauna of the lower moa river forest, state of Acre, Brazil. Zoologia 32(2): 93–108. http://dx.doi.org/10.1590/S1984-46702015000200001.

[B40] Neckel-Oliveira S Galatti U Faveri SB (2013) Ecological correlates in Brazilian Amazonian anurans: implications for conservation. Amphibia-Reptilia 34: 217–232. https://doi.org/10.1163/15685381-00002890

[B41] Ouboter PE Jairam R (2012) Amphibians of Suriname. Brill Academic Publisher, 388 pp.

[B42] Pantoja DL Fraga R (2012) Herpetofauna of the Reserva Extrativista do Rio Gregório, Juruá Basin, southwest Amazonia, Brazil. Check List 8(3): 360–374.

[B43] Peloso PLV (2010) A safe place for amphibians? A cautionary tale on the taxonomy and conservation of frogs, caecilians, and salamanders in the Brazilian Amazonia. Zoologia 27(5): 667–673. https://doi.org/10.1590/S1984-46702010000500001

[B44] Peloso PLV Sturaro MJ Forlani MC Gaucher P Motta AP Wheeler WC (2014) Phylogeny, Taxonomic Revision, and Character Evolution of the Genera *Chiasmocleis* and *Syncope* (Anura, Microhylidae) in Amazonia, with Descriptions of Three New Species. Bulletin of the American Museum of Natural History 386(1): 1–112. http://dx.doi.org/10.1206/834.1

[B45] Pereira-Júnior AP Costa-Campos CE Araújo AS (2013) Composição e diversidade de anfíbios anuros do campus da Universidade Federal do Amapá. Biota Amazônia 3(1): 13–21. http://dx.doi.org/10.18561/2179-5746/biotaamazonia.v3n1p13-21

[B46] Piatti L Amaro PMO Araújo JFJ Sanches VQA Bernarde PS (2012) Anurans of a disturbed area in Jarú, Rondônia, Brazil. Check List 8(1): 083–087.

[B47] Prudente ALC Sturaro MJ Travassos AEM Maschio GF Santos-Costa MC (2013) Anurans of the Urucu Petrol Basin, municipality of Coari, State of Amazonas, northern Brazil. Check List 9(3): 601–606.

[B48] Queiroz SS Silva AR Reis FM Lima JD Lima JRF (2011) Anfíbios de uma área de castanhal da Reserva Extrativista do Rio Cajari, Amapá. Biota Amazônia 1(1): 1–18. https://doi.org/10.18561/2179-5746/biotaamazonia.v1n1p1-18

[B49] Ramalho WP Andrade MS Matos LRA Vieira LJS (2016) Amphibians of varzea environments and floating meadows of the oxbow lakes of the Middle Purus River, Amazonas, Brazil. Biota Neotropica 16(1): e20150093. https://doi.org/10.1590/1676-0611-BN-2015-0093

[B50] Rodríguez L Martinez JL Coloma LA Ron S Azevedo-Ramos C Castro F Rueda JV Cisneros-Heredia D Hoogmoed M Gascon C (2004) *Pristimantis ockendeni* The IUCN Red List of Threatened Species 2004. e.T56803A11534464. http://dx.doi.org/10.2305/IUCN.UK.2004.RLTS.T56803A11534464.en [accessed on 23 February 2018]

[B51] Segalla MV Caramaschi U Cruz CAG Garcia PCA Grant T Haddad CFB Langone P (2016) Brazilian amphibians – List of species. http://www.sbherpetologia.org.br [accessed on 15 July 2016]

[B52] Silva e Silva YB Costa-Campos CE (2014) *Scinax garbei* (Miranda-Ribeiro, 1926) (Amphibia: Anura: Hylidae): Distribution extension for Brazilian Amazonia and first record in the state of Amapá. Check List 10(2): 448–449. http://www.checklist.org.br/getpdf?NGD089-13

[B53] Silva e Silva YB Costa-Campos CE (2016) *Hyalinobatrachium iaspidiense* (Ayarzaguena, 1992) (Anura: Centrolenidae): first record in Amapá state, Brazil and geographic distribution map. Check List 12(2): 1849, doi: http://dx.doi.org/10.15560/12.2.1849

[B54] Silva e Silva YB Costa-Campos CE Valentim DSS Melo-Furtado MF (2015) *Pristimantis ockendeni* (Carabaya Robber Frog). Brazil, Amapá. Herpetological Review 46(1): 58.

[B55] Silvano DL Segalla MV (2005) Conservação de anfíbios no Brasil. Megadiversidade: Desafios e oportunidades para a conservação da biodiversidade no Brasil 1(1): 79–86.

[B56] Vaz-Silva W Oliveira RM Gonzaga AFN Pinto KC Poli FC Bilce TM Penhacek M Wronski L Martins JX Junqueira TG Cesca LCC Guimarães VY Pinheiro RD (2015) Contributions to the knowledge of amphibians and reptiles from Volta Grande do Xingu, northern Brazil. Brazilian Journal of Biology 75(3): S205–S218. https://doi.org/10.1590/1519-6984.00814BM 10.1590/1519-6984.00814BM 26691094

[B57] Venâncio NM Souza MB (2016) Anfíbios do Parque Ambiental Chico Mendes, Rio Branco – Acre, Brasil. Biotemas 29(1): 85–95. https://doi.org/10.5007/2175-7925.2016v29n1p85

[B58] Waldez F Menin M Vogt RC (2013) Diversidade de anfíbios e répteis Squamata na região do baixo rio Purus, Amazônia Central, Brasil. Biota Neotropica 13(1): 300–316.

[B59] Zar JH (1999) Biostatistical analysis. Prentice Hall, New Jersey, 663 pp.

